# Electrochromic Efficiency in A*_x_*B_(1−*x*)_O*_y_*-Type Mixed Metal Oxide Alloys

**DOI:** 10.3390/ijms26083547

**Published:** 2025-04-10

**Authors:** Zoltán Lábadi, Noor Taha Ismaeel, Péter Petrik, Miklós Fried

**Affiliations:** 1 Institute of Technical Physics & Materials Science, HUN-REN Centre for Energy Research, Konkoly-Thege Rd. 29-33, 1121 Budapest, Hungary; labadi.zoltan@ek-cer.hu (Z.L.); petrik.peter@ek.hun-ren.hu (P.P.); 2 Doctoral School on Materials Sciences and Technologies, Óbuda University, 1034 Budapest, Hungary; noor.t@ilps.uobaghdad.edu.iq; 3 Institute of Laser for Postgraduate Studies, University of Baghdad, Baghdad 10070, Iraq; 4 Department of Electrical Engineering, Institute of Physics, Faculty of Science and Technology, University of Debrecen, Bem tér 18, 4026 Debrecen, Hungary; 5 Institute of Microelectronics and Technology, Óbuda University, Tavaszmezo Str. 17, 1084 Budapest, Hungary

**Keywords:** reactive sputtering, mixed metal oxides, coloration efficiency, electrochromic materials

## Abstract

Electrochromic materials have a wide range of energy-effective applications, such as in mirrors, smart windows, automobile sunroofs, and display devices. The electrochromic behavior of mixed metal oxides is focused on in this review. Extra heat absorbed by buildings is one of the major problems in our modern era, so electrochromic films have been used as components of smart windows to reduce heat absorption through glass windows. Transition metal (W, V, Ti, Mo, and Ni) oxides are considered popular electrochromic materials for this purpose. Smart windows consist of electrochromic material layers (such as metal oxide layers) and solid electrolytes sandwiched between transparent conductive layers. Few publications have studied the use of mixtures of different metal oxides as electrochromic materials. This study focuses on the results of investigations of such multicomponent materials, such as the effects on the electrochromic properties of mixed metal oxides and how they contrast with pure metal oxides. Reviewing these papers, we found WO_3_- and MoO_3_-based mixtures to be the most promising, especially the magnetron-sputtered, amorphous WO_3_(40%)–MoO_3_(60%) composition, which had 200–300 cm^2^/C coloration efficiency. The mixed oxide materials reported in this review have room for development (and even commercialization) in the oxide-based electrochromic device market.

## 1. Introduction

Ventilation, air conditioning, and heating in constructions account for 30–40% of the world’s energy usage [1]. Up to 40% of the energy deficiency in buildings has been reduced by enhancements in the optical properties and thermal absorption of windows [2]. Therefore, developing technologies such as electrochromic windows can be the best choice to reduce energy usage in buildings because, when applied to DC bias, changing the light-transmitting properties makes controlling the transparency of windows easier [3,4,5]. Even in commercial constructions, controlling sunlight transmission can reduce lighting and energy expenses by 20–50% [1,6,7]. Metal oxide layers are the active components of electrochromic windows [3,8,9,10,11], as well as organic films, which present electrochromism, a phenomenon that makes materials change their optical properties after a charge injection or extraction [3,11].

Historically, the first group of materials found to display electrochromic properties was metal oxides. Electrochromic (EC) oxides in which cathodic coloration occurs include WO_3_, TiO_2_, MoO_3_, SnO_2_, and Ta_2_O_5_, while anodic coloration occurs in NiO, CrO, MnO_2_, Co_3_O_4_, and V_2_O_5_ [12,13,14,15,16,17,18,19]. These inorganic EC materials have high chemical and thermal stability, device durability, and cycling stability. However, these materials also have inherent drawbacks, i.e., they provide slower response times, they have mechanical rigidity, and, most importantly, their coloration is limited to a narrow spectral range. Their rigidity limits their use in flexible devices and roll-to-roll technologies, while their spectral properties limit their application in color displays.

Apart from the transition of metal oxides, the application of organic small-molecule EC materials has to be mentioned. Viologen is one of the first examples to have been successfully commercialized in Boeing 787 aircraft windows. Other small-molecule EC materials include terephthalate and isophthalate derivatives and other dye molecules. Small-molecule materials typically provide more precise color control and a faster response time, making them suitable for applications such as high-resolution displays. The synthesis of these compounds is relatively straightforward, and their color and performance can be easily adjusted by modifying their molecular structure. However, the stability and durability of these materials are often lacking, especially when exposed to long-term ultraviolet irradiation and oxygen in the air.

The newest generation of EC materials includes organic polymers. A wide range of conjugated polymers (polythiophene, polypyrrole, polyaniline, polyindole, polyfuran, and polycarbonazole) represent a significant class of EC materials. These polymer materials possess good processability and are available at a low cost. However, their sensitivity to environmental variables such as humidity and temperature poses challenges to their durability and stability. 

Demand–supply factors and pricing depend on the accuracy of preparation methods for material mixes. (Physical deposition methods are promising for the market because these preparation methods are highly accurate.) These mixed metal oxides show enhanced electrochromic behavior and enhanced energy savings compared with pure materials. Electrochromic systems are also utilized in energy storage applications. These combinatorial methods can help identify more efficient advanced functional materials for different applications, such as micro-, nano-, and optoelectronics; energy converters (solar cells) or optical sensor systems; architectural glazing; high-contrast displays; sunroofs; sunglasses; and smart windows.

To scale up production, a variety of preparation methods of mixed metal oxides have recently been recognized to provide samples that are considered more economical and environmentally friendly. To examine the utilization of mixed metal oxides in different commercial products, effort has been put into determining cost-effective and scalable synthesis protocols. For example, reactive sputtering is well known as a scalable method; however, it can be relatively expensive to use large vacuum systems. However, this can produce highly accurate samples. To create larger samples at lower expenses, different preparation methods, such as sol–gel, can be used, sacrificing part of the accuracy. We must always consider safety standards when working with metal oxides and not exceed the risk limits of the excessive handling of such materials without adhering to safety conditions. Therefore, one should avoid toxic materials or excessively deal with carcinogenic oxidizing materials. Metal oxides, which are ubiquitous in industrial processes and consumer products, are known to leach into water bodies, posing a significant threat to aquatic ecosystems. Additionally, synthetic dyes, which are extensively used in various industries, can persist in water systems and exhibit complex chemical behavior.

Electrochromic phenomena occur after applying a DC electric current, and the color of the material is changed. Transition metal oxide films (molybdenum and tungsten) are considered the most popular and most studied materials in this field. These are used in solid-state electrochromic devices in the electrolyte layers (Ta_2_O_5_), which are sandwiched between transparent conducting electrodes (TCEs), electrochromic materials (WO_3_, MoO_3_), and charge storage. 

Electrochromic materials have several key parameters that significantly influence their performance and suitability for different applications. The most important ones are the following:

- The coloration efficiency measures the amount of optical change per unit of charge injected into the material. A higher coloration efficiency indicates a more efficient use of energy and can lead to lower power consumption in devices.
- Optical modulation refers to the change in optical properties (transmittance, reflectance, and absorbance) when a material undergoes a redox reaction. High optical modulation is crucial for achieving significant color changes and achieving desired functionalities, such as dimming windows or creating displays.
- Response time is the speed at which a material changes color in response to an applied voltage. Faster response times are generally desirable for applications such as dynamic displays or rapidly adjusting window tints.
- Cycling stability refers to the ability of a material to maintain its electrochromic properties over repeated cycles of coloration and bleaching. Long-term stability is essential for practical applications to ensure durability and longevity.
- Durability encompasses various factors, including resistance to degradation from environmental factors such as moisture, temperature, and UV radiation. Durable materials are necessary for long-lasting performance in real-world applications.
- The color range is the range of colors that a material can achieve and is important for aesthetic and functional considerations. Materials that can achieve a wide range of colors offer greater versatility in applications.
- Transparency in the bleached state is crucial for applications such as smart windows. High transparency is needed in the bleached state to allow maximum light transmission when not in use.
- The operating voltage is the voltage required to induce color changes. It should be low to minimize power consumption and ensure compatibility with various power sources.

These parameters are interconnected and often influence each other. For example, optimizing fast response times may sometimes come at the expense of cycling stability. Researchers and engineers must carefully consider these parameters and their trade-offs when developing and selecting electrochromic materials for specific applications. These parameters depend on the structural, compositional, and morphological characteristics, as well as growth parameters and deposition techniques.

The overall performance of an electrochromic device is determined by four major physical characteristics: coloration efficiency (CE), optical contrast, switching time, and cycling stability. High-performance electrochromic devices (ECDs) must have a high CE, high contrast, fast switching time, long cyclic stability, and high efficiency to meet the market requirements.

Of these four requirements, the CE is a key parameter in determining the performance of an electrochromic device. This indicator shows the change in optical absorption per unit of charge. It is usually expressed as the change in optical density per unit of charge passed per unit area (cm^2^/C). A high coloration efficiency means that a relatively small, injected charge can initiate a significant color change, directly impacting energy efficiency.

The contrast ratio is defined as the ratio of visible light transmission in the bleached vs. the colored state. A higher contrast ratio means more visible change from the darkest to the lightest state, which is especially important for ECDs.

The switching time of an ECD refers to the time needed for the device to achieve 90% of its maximum optical change when transformed from a bleached (transparent) state to a colored state or vice versa. This parameter is also known as the response time or, alternatively, the coloring/bleaching time. Typically, EC materials or devices that exhibit faster response times are preferred. Short response times mean rapid color changes, making them critical for devices that require rapid changes.

Cycling stability and lifetime parameters describe the stability and degradation of EC materials in long-term use. Cycling stability refers to the ability of a material to maintain its properties through prolonged coloring and bleaching cycles. The lifetime is given by the number of coloring and bleaching cycles that a material undergoes before it degrades to a predetermined level. High durability and long life are essential for commercial applications to ensure long-term reliability and cost-effectiveness of products.

The stability of commercialized ECDs can vary depending on the materials used and the environmental conditions to which they are exposed. Electrochromic polymers (ECPs) can degrade quickly in the presence of UV light and oxygen. Exposure to sunlight, humidity, and temperature changes can also affect the stability of ECDs. Devices designed for indoor use tend to have longer lifespans than those exposed to outdoor conditions.

Another important question is whether cycling stability if the CE is maximized. The ability of an ECD to maintain performance over many cycles of switching between states is important. Advancements in materials and encapsulation techniques are improving the stability of ECDs, making them more viable for long-term applications in smart windows, displays, and wearable technology. The typical lifespan of smart films can be extended to 10–20 years with proper care and preparation methods. Factors such as exposure to the elements, the quality of the substrate glass, and regular maintenance all play a part in determining a device’s longevity and toughness. The following is a comparison of the average lifespan of different types of window films: solar control film—10–15 years; decorative film—5–10 years; privacy film—5–10 years; and safety and security film—10–15 years.

According to the U.S. Department of Energy, replacing old single-paned windows with energy-efficient replacements should save 7% to 15% in energy costs, or roughly USD 71 to USD 501 annually. So, it is worth the effort and the expected challenges in fitting with current window systems. Windows and doors can be made smart without replacing them; it is only necessary to fit them with smart sensors, locks, or mechanisms that can be connected to one’s home network, usually via a smart hub. This allows them to be operated with an app on one’s smartphone or tablet. There are also windows and doors with integrated smart systems.

Measuring the CE of electrochromic films is possible with different methods.

Chronoabsorptometry and chronocoulometry: This method involves measuring the change in optical absorbance (Δ*A*) at a specific wavelength (*λ*) while simultaneously recording the charge (*Q*) passed during the electrochemical reaction. 

The coloration efficiency is then calculated using the following formula:

CE = OD/Δ*Q* (1)

where Δ*Q* is the inserted/extracted charge density, and the optical density (OD) is the film thickness multiplied by the absorption coefficient.

Differential coloration efficiency measures the derivative of the optical density with respect to the unit of inserted charge. This is useful for materials such as amorphous metal oxides, where the coloration efficiency varies with the level of ion intercalation.

In situ colorimetric methods track changes in color coordinates during the electrochemical process. This can provide detailed information about the hue, saturation, and luminance changes in an electrochromic film.

Spectral measurements: By measuring the transmittance or reflectance spectra of an electrochromic film before and after coloration, one can determine the efficiency based on the changes in optical properties. This method is often combined with electrochemical measurements to provide a comprehensive analysis. 

The optical parameters and compositions can be determined and mapped using spectroscopic ellipsometry (SE). Rutherford backscattering spectrometry (RBS) and scanning electron microscopy (SEM) with energy-dispersive X-ray spectroscopy (EDS) are capable of checking the composition of the layers. A variety of other methods, including X-ray diffraction (XRD), transmission electron microscopy (TEM), and atomic force microscopy (AFM), can be used to investigate the microstructure of sample layers.

The EC effect can be more obvious in mixed oxides due to potential electron transitions between two sets of electrons. In addition, the EC properties of mixed metal oxides are better than those of pure oxides because, in mixed metal oxide layers, the electrochromic effectiveness can be higher, and mixing metal atoms with different diameters in the layers can enhance the CE. Despite this, relatively few studies have focused on the possible enhancement of EC parameters in mixed-oxide-type films. The aim of this review is to synthesize the results of investigations of this type of mixed oxide layer.

## 2. Overview

### 2.1. “Simple” Metal Oxides

Tungsten oxide (WO_3_) is considered the most widely studied oxide for electrochromism; films of this material have been prepared using several different methods. The traditional preparation methods for thin films include the following: chemical methods (sol–gel deposition, spin coating, the Langmuir–Blodgett technique, chemical bath deposition, etc.), electrochemical methods (anodization, plating), and chemical and physical vapor deposition; see [10] and the references therein. Examples of physical vapor deposition include thermal evaporation [20,21,22,23,24], sputtering [25,26], and pulsed laser deposition [27,28].

The chemical methods that have been described include chemical vapor deposition [29,30,31] and associated spray pyrolysis [32,33,34,35,36]. The fabrication of W oxide films has been described using chemical methods of investigation [37,38,39]. Most cases of a coloration efficiency (CE) of more than 60 cm^2^/C were found in the red region [10]. Electrodeposition [40,41], anodic oxidation [42,43,44], and electrodeposition [45] have been described by other investigators. Prepared films were described in evaporation studies that presented the electrochromic behavior of Mo oxide (MoO_3_) in comparison with W oxide, and the CE was measured to be 34 cm^2^/C at 630 nm [46,47]. Chemical vapor deposit was described in [48]; other studies have used wet chemical techniques [15,49], and electrodeposition was presented in [50]. Ti oxide (TiO_2_) was investigated for its electrochromic (EC) properties when prepared with different methods, such as sputtering [51], chemical vapor deposition [52], spray pyrolysis [53,54], various wet chemical techniques [55,56,57], and anodization [58,59,60]. The CE of TiO_2_ films that were deposited using reactive DC magnetron sputtering was approximately ∼25 cm^2^/C. Various chemical techniques have verified that such films can exhibit the same CE values as those of TiO_2_ films [51]. Nb oxide films were prepared chemically, and they presented electrochromic results [61,62,63,64]. The CE of Ta oxide films was 10–15 cm^2^/C, and this result was considered good for bleaching/coloration cycles; the value of 79.8% demonstrated the high reversibility, and the large ion-diffusion coefficient was 2.35 × 10^−8^ cm^2^/s [65]. A technique known as r.f. reactive sputtering was presented in [66]. Titanium oxide (TiO_2_) was prepared using the thermionic vacuum arc method in [67].

### 2.2. Mixed Oxides

#### 2.2.1. TiO_2_–WO_3_

In nano-array films, a notable enhancement of EC properties was investigated by Cai et al. [68], and the EC results for TiO_2_ and WO_3_ core–shell nanorod arrays indicated that they are promising materials because a larger surface area for charge-transfer reactions was obtainable for ion diffusion. They were prepared using a combination of electrodeposition and hydrothermal methods. Na_2_WO_4_ salt was dissolved in deionized water at a concentration of 12.5 mM. Sodium tungstate was mixed into the solution with hydrogen peroxide while preserving a concentration ratio of 3. Fluorine-doped tin oxide (FTO) glass was coated with a deposition electrode as a TiO_2_ nanorod array. An enhancement of EC properties was achieved in the porous space surrounded by the nanorod arrays and the core/shell nanorod array structure. The following significant improvements were achieved: fast switching speed (2.4 s and 1.6 s), high CE (67.5 cm^2^/C at 750 nm), magnificent cycling behavior (65.1% after 10,000 cycles), and wonderful consequences of optical modulation (38.4% at 10 μm, 70.3% at 1800 nm, and 57.2% at 750 nm).

The spray pyrolysis technique was investigated by Patil et al. [69]. They deposited TiO_2_-doped WO_3_ thin films at 525 °C on conducting glass substrates coated with FTO. The first materials that were used for the deposition of TiO_2_-doped WO_3_ thin films were titanyl acetylacetonate (C_10_H_14_O_5_Ti) and tungsten trioxide (WO_3_). At 80 °C, an ammonium tungstate solution was prepared by dissolving WO_3_ powder in liquid ammonia. Titanyl acetylacetonate (C_10_H_14_O_5_Ti) powder was separately dissolved in methanol at room temperature. To form a homogeneous 100 ml precursor solution at pH = 9, the two solutions were mixed in contrary volume percentages. The dopant volume percentage of TiO_2_ varied between 13% and 38% *v/v*. The thin film samples were transparent, strongly stuck to the substrates, and were uniform. Pour dominants could not be formed (for percentages of TiO_2_ doping greater than 38% in a homogeneous WO_3_ solution). With the help of chronocoulometry (CC), cyclic voltammetry (CV), and chronoamperometry (CA) techniques, during the study of the electrochemical properties of TiO_2_-doped WO_3_ thin films, the researchers concluded that TiO_2_ doping enhances the electrochromic performance of WO_3_, and the samples revealed increasingly high reversibility in accordance with the doping concentrations of TiO_2_.

**Figure 1:**
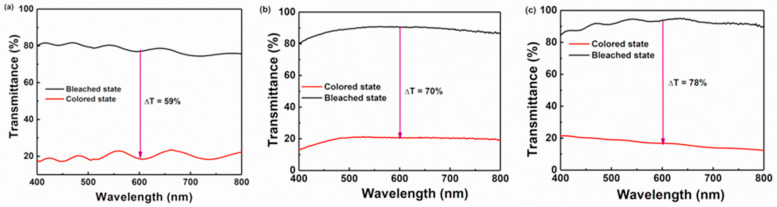
Bleached and colored transmittance spectra of (**a**) TiO_2_, (**b**) deposited WO_3_/TiO_2_, and (**c**) annealed WO_3_/TiO_2_ films [70].

The low-temperature preparation of WO_3_/TiO_2_ films was investigated by Dhandayuthapani et al. [70] by synthesizing deposition techniques (nebulized spray and chemical bath). The WO_3_ layer impacted on the compositional, structural, electrochemical, and morphological characteristics of the TiO_2_ films. The current density of the TiO_2_ films was beneficially improved by the deposition of WO_3_ nanoplates on the TiO_2_ layer. Figure 1 presents the results of the electrochemical investigation of the annealed WO_3_/TiO_2_ films. The reversibility was 77.2%, the CE was 128.3 cm^2^/C, and the optical modulation (Δ*T*) was 78%. A fast response of 6 s for bleaching and coloration was indicated with magnificent durability for 1000 cycles. The enhancement of EC behavior was caused by the facilitation of faster charge transport, the interconnected nanoplate bundles that provided more charges, and the complementarity of the WO_3_–TiO_2_ layers.

**Figure 2:**
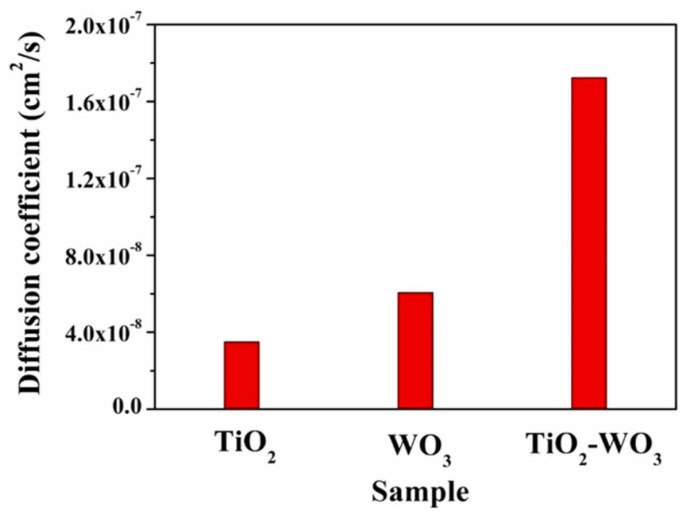
Comparison of the diffusion coefficients of TiO_2_–WO_3_, TiO_2_, and WO_3_ films [71].

The assembly of titanium dioxide nanorods (TiO_2_)/hybrid tungsten oxide (WO_3_) thin films was investigated by Ashok Reddy et al. [71], in addition to the impact of nanostructures on the electrochromic characteristics of the films. Using the sputtering deposition method, enhanced WO_3_ films were coated with TiO_2_ nanorod film. Under diverse partial pressures of oxygen and at a temperature of 400 °C, WO_3_ thin films were deposited on fixed substrates. Using a hydrothermal process, tungsten nanorods were grown on a glass substrate that was coated with fluorine-doped tin oxide (FTO). UV–visible spectrometry, Raman spectrometry, XPS, XRD, and cyclic voltammetry were used for electrochemical and material analyses of the films. Figure 2 presents the results. The TiO_2_ nanorods/hybrid WO_3_ films revealed impressive electrochemical characteristics relative to the diffusion coefficient of 1.8 × 10^−7^ cm^2^/s, which surpassed that of pure (WO_3_ and TiO_2_) nanorods. The optimized coloration efficiency of the enhanced WO_3_ films was attributed to their large active surface area, which preferred the insertion of H^+^ ions into the films.

**Figure 3:**
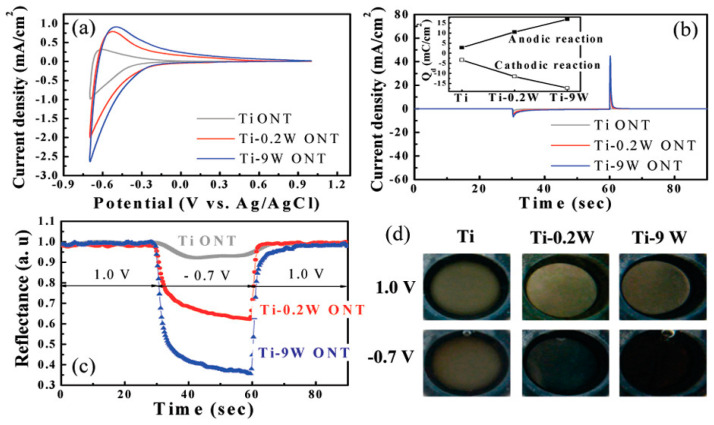
(**a**) Cyclic voltammograms of oxide nanotube (ONT) layers on Ti, Ti-0.2W, and Ti-9W performed between −0.7 and 1.0 V with a scan rate of 50 mV in 0.1 M HClO_4_ electrolyte; (**b**) current density−time curves acquired through pulse potential measurement applied between −0.7 and 1.0 V with a duration of 30 s; (**c**) in situ reflectance curves of Ti, Ti-0.2W, and Ti-9W ONTs obtained during potential pulsing applied between 1.0 and −0.7 V; (**d**) optical images of the electrochromic effects of the different nanotube surfaces during polarization cycling between 1 and −0.7 V. The inset of (**b**) shows the integrated charge density (*Qd*) for the samples. Reprinted (adapted) with permission from [72]. Copyright 2008 American Chemical Society.

Under special electrochemical conditions, regular and homogeneous arrays of TiO_2_−WO_3_ nanotubes were investigated by Nah et al. [72], and they concluded that such nanotubes can be layered through the anodization of Ti alloys in an ethylene glycol/fluoride-based electrolyte. Using anodization at 120 V in a solution of ethylene glycol with 0.2 M HF, they sputtered nanotube films on various substrates (Ti-9 at% W (Ti-9W) and Ti, Ti-0.2 at % W (Ti-0.2W)). The growing time was controlled to compare the thickness of the layers. A thickness of 85−95 nm and a tube diameter of 1.1−1.2 μm were achieved for the ordered oxide nanotube layers. The improvements of the enhanced EC reactions and properties due to small amounts of WO_3_ (such as 0.2 at%) in these mixed oxide nanotube structures, as well as the onset potential, contrast, and cycling stability of nanotube-layer-based devices, are shown in Figure 3.

#### 2.2.2. SnO_2_–WO_3_

Using a dip-coating method, Nd–Mo-co-doped SnO_2_/α-WO_3_ electrochromic materials were deposited by Goei et al. [73]. The Nd–Mo-co-doped SnO_2_/α-WO_3_ ECs revealed up to 90% visible light transparency at λ = 600 nm in comparison with conventional SnO_2_/α-WO_3_ ECs after up to 1000 volatile cyclic trials, and there was a 59% drop in electrochromic functionality with respect to the undoped device after up to a test with 1000 reversible cycles. Moreover, these doped samples displayed shorter switching time (31% of the undoped) and high coloration efficiency (~200 cm^2^/C). The authors claimed that these improved characteristics related to the addition of Nd–Mo co-dopants, which restricted the trapping of Li+ ions within the α-WO_3_ framework and decreased the extent of crystallization of the α-WO_3_ layer.

Kim et al. [74] added antimony-doped tin oxide nanoparticles (ATO NPs) to WO_3_ EC films at three different concentrations: 0, 0.6, 1.2, and 2.4 wt%. The concentration of 1.2 wt% was found to be optimal, as the WO_3_ EC film with ATO NPs displayed better EC performance in terms of both the CE value (48.2 cm^2^/C) and the switching time (2.4 s for the bleaching time and 5.4 s for the coloration time). The authors concluded that the large bandgap of ATO NPs widened the transmittance modulation band of the EC layer, which helped to increase the CE value. A further component of the enhancement of the EC performance was found to be the improved electrical conductivity caused by the evolution of preferable electron tracks due to the good dispersion of ATO NPs in the WO_3_ film. 

**Figure 4:**
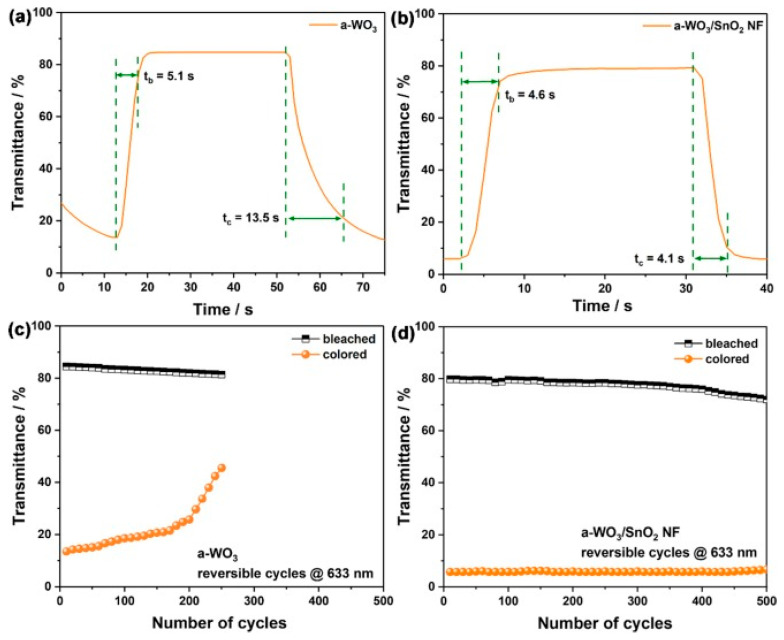
Variation curves at 633 nm when electrochromic windows composed of α-WO_3_ (**a**) and α-WO_3_/SnO_2_ NF (**b**) were switched from the bleached to the colored state. The response times are indicated. Durability tests of electrochromic windows composed of α-WO_3_ (**c**) and α-WO_3_/SnO_2_ NF (**d**) were cycled 500 times at 633 nm [75].

In a recent work, Wei Wei et al. [75] showed an electrochromic window that had adequate characteristics, particularly in the NIR region. The electrochromic layer was made up of hierarchical amorphous WO_3_/SnO_2_ nanoflake arrays that were prepared by combining hydrothermal and ultraviolet photodeposition processes. UV–vis–NIR transmittance spectra, photothermal maps, and electrochemical tests (CV, CA, and EIS) showed that the hierarchical film displayed improved electrochromic characteristics, including a fast response time, proper cycling durability, and high coloration efficiency. The hierarchical amorphous WO_3_/SnO_2_ nanoflake arrays also had adequate transmittance modulation in the NIR region. The NIR contrasts reached 65.7% and 66.5% at 1200 and 1600 nm, respectively. The visible contrast was 73.5% at 633 nm; see Figure 4.

#### 2.2.3. WO_3_–NiO

S. V. Green [76] investigated the structure of an electrochromic Ni*_x_*W_1−*x*_ oxide thin film in which 0 < *x* < 1. Reactive DC magnetron co-sputtering was applied to deposit the thin layers from one Ni and one W metal target. Furthermore, the Ni oxide was deposited by adding water vapor to the sputtering gas. Structural characterization of all layers was accomplished using X-ray diffraction, *X*-ray photoelectron spectroscopy, Raman spectroscopy, and Rutherford backscattering spectrometry. The nanostructures of different films were studied with ellipsometry using effective medium approximation. The electrochemical and optical properties were characterized using cyclic voltammetry and spectrophotometry in 1 M lithium perchlorate in propylene carbonate (Li-PC).

**Figure 5:**
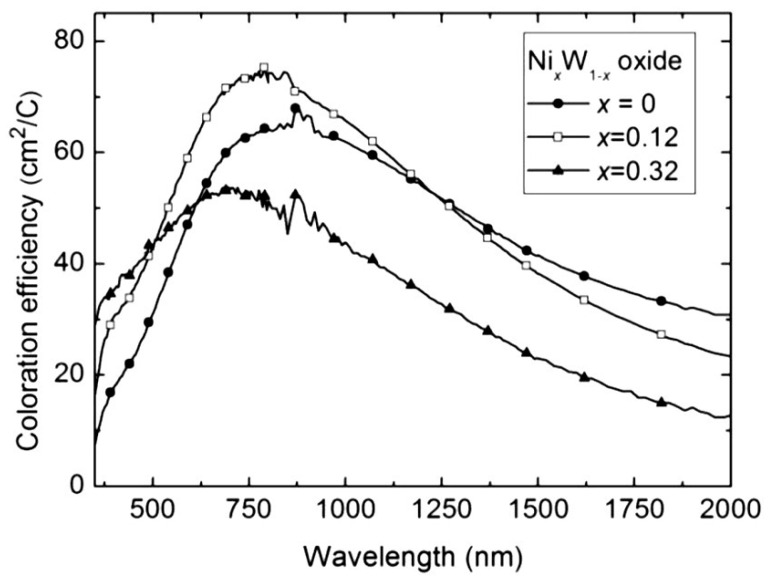
Spectral coloration efficiency for W–Ni oxide films with the compositions shown [77].

Samples with high (over 85%) Ni content were found to be polycrystalline, while all other films were amorphous. W-rich samples (Ni content below 50%) consisted of a blend of W oxide and NiWO_4_ phases, while the Ni-rich (over 50%) samples were made of hydrated Ni oxide and NiWO_4_ phases. Samples with 0 < *x* < 0.3 exhibited electrochromic properties matching those of W oxide, and films with 0.7 < *x* < 1 functioned as Ni oxide. For 0.4 < *x* < 0.7, no optical change was observed. The sample behaved as an optically inert intercalation material at the boundary of cathodic electrochromic and non-electrochromic characteristics, i.e., *x*~0.4. The addition of Ni to W oxide enhanced the coloration efficiency. Spectral coloration efficiency curves are shown for selected compositions of W–Ni oxide films in Figure 5 [77]. The transmittance changes were 0.45 and 0.15 for the W-rich and Ni-rich samples, respectively.

**Figure 6:**
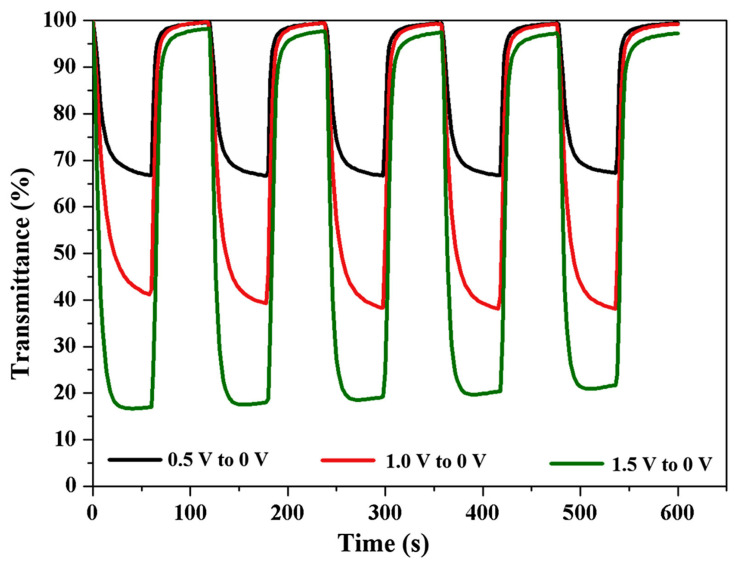
EC performance of a NiO/WO_3_ ECD (ITO) showing stability at different voltages [78].

Rakibuddin et al. [78] synthesized NiO using a cost-effective sol−gel spin-coating process. The as-prepared NiO was used as an electrochromic anodic layer and deposited onto a transparent conductive electrode (indium tin oxide (ITO) or flexible silver nanowires (AgNWs)) through sol–gel spin coating and low-temperature annealing. The deposition methods were optimized to achieve better EC characteristics. NiO/ITO displayed high transmittance variance (Δ*T* = ~84%) at 700 nm with applied potentials of −3.0 and 0 V. The stability and transmittance variance of NiO/ITO were significantly improved in the presence of a WO_3_ cathodic electrode at lower applied voltages of 1.5 to 0 V; see Figure 6. The flexible NiO–WO_3_ device reached a transmittance variation of ~38% at 700 nm with applied potentials of 2.0 and 0 V, and the EC performance was preserved.

#### 2.2.4. WO_3_–Ag

Najafi-Ashtiani et al. [79] used physical vapor deposition (PVD) to prepare electrochromic tungsten oxide thin films on fluorine-doped tin oxide (FTO)-coated glass substrates. In order to achieve changes in the surface morphology, they made a PVD preparation at angles of 0° and 75°. In combination with the PVD method, powdered Ag nanoparticles were used to dope the surface of the WO_3_ thin layers. The EC thin layers were annealed to diffuse the Ag nanoparticles into the layer. Indirect transitions were reported in the bandgap of the WO_3_–Ag thin films. Using cyclic voltammetry and visible-light transmittance data, the EC characteristics of the WO_3_–Ag thin films were studied to evaluate the effect of the surface morphology. Higher surface roughness, clear optical modulation (40.59% at 632.8 nm), and high coloration efficiency (90.2 cm^2^/C at 632.8 nm) were detected for the second sample, which was prepared at an angle of 75° (see Equations (2) and (3) and Table 1).

CE = ΔOD/(*Q*/*A*) (2)

ΔOD (*λ*) = log *T_b_*/*T_c_* (3)

where OD is the optical density, *Q*/*A* is the injected electronic charge per unit area, and *T_c_* and *T_b_* are the transmission of thin layers at λ = 632.8 nm in the colored and bleached states, respectively.

**Figure 7:**
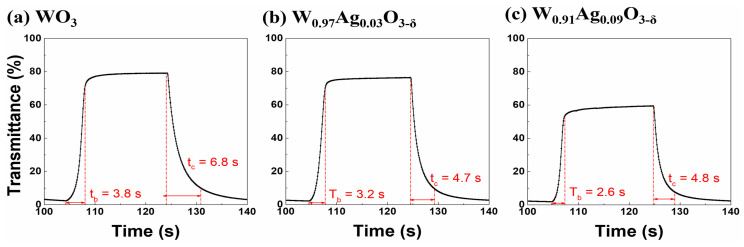
Transmittance switching response of (**a**) WO_3−δ_, (**b**) W_0.97_Ag_0.03_O_3−δ_, and (**c**) W_0.91_Ag_0.09_O_3−δ_ thin films grown at room temperature on ITO-coated glass substrates [80].

Recently, Park et al. [80] reported the structural, optical, and electrochemical properties of Ag nanoparticles contained in thin electrochromic WO_3_ layers with various concentrations. Room temperature-sputtered thin films using composite targets with a low Ag concentration showed an irregular distribution of small nanoparticles, while with a higher Ag concentration, the particles were merged, leading to the formation of a single or polycrystalline phase. They measured surface plasmon resonance peaks in the absorption spectra of the Ag–WO_3_ composite layers, indicating the development of metallic nanoparticles. The presence of these nanoparticles was also reaffirmed via high-resolution transmission electron microscopy. The thin W_0.91_Ag_0.09_O_3−δ_ layer had a faster switching time with a higher coloration efficiency of 66.52 cm^2^/C than the WO_3−δ_ thin layer, which had a value of 58.68 cm^2^/C. However, the transmittance modulation in the W_0.91_Ag_0.09_O_3−δ_ thin film was worse than that in the other films; see Figure 7. Furthermore, the W_0.91_Ag_0.09_O_3−δ_ thin films were black in the colored state, whereas the pure WO_3−δ_ and W_0.97_Ag_0.03_O_3−δ_ thin films were Prussian blue, suggesting the achievement of color neutrality through the incorporation of Ag nanoparticles.

#### 2.2.5. V_2_O_5_–WO_3_

Patil et al. [81] prepared mixed V_2_O_5_–WO_3_ layers on FTO-coated glass substrates at 400 °C using a novel pulsed spray pyrolysis technique (PSPT). Aqueous solutions of equimolar vanadium chloride and ammonium tungstate were mixed in three different volume proportions (5%, 10%, and 15%) for the preparation of V_2_O_5_–WO_3_ thin-film layers. The optical, morphological, structural, and electrochemical properties of V_2_O_5_–WO_3_ thin films were studied through cyclic voltammetry, XRD, FTIR, SEM, chronocoulometry, and chronoamperometry. Their results showed that the electrochemical properties of V_2_O_5_ were modified by mixing it with WO_3_. All of the films showed cathodic electrochromism in a lithium-containing electrolyte (0.5 M LiClO_4_ + propylene carbonate (PC)). The electrochemical durability of the samples was studied and found to be stable for up to 1000 cycles. A maximum coloration efficiency (CE) of about 49 cm^2^/C was measured for the V_2_O_5_ film mixed with 15% WO_3_. 

**Figure 8:**
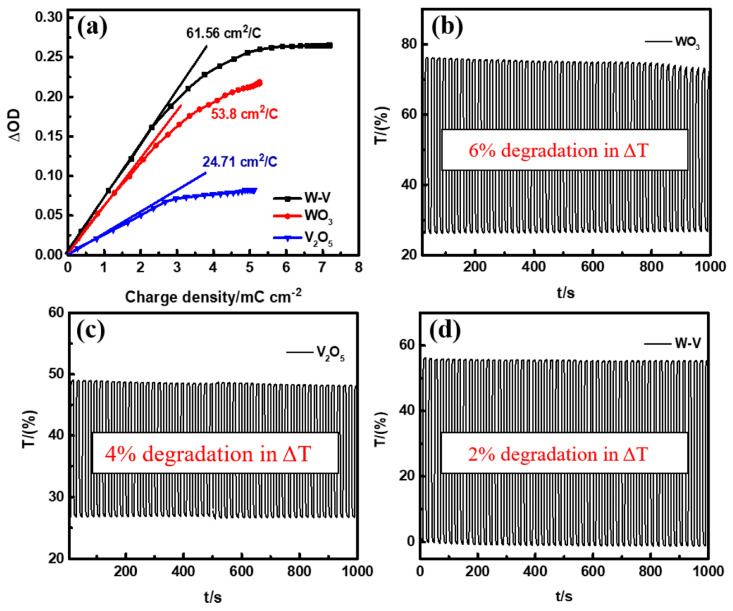
Variations in the change in optical density (ΔOD) vs. charge density for WO_3_, V_2_O_5_, and W–V thin films (**a**). In situ optical responses of WO_3_ (**b**), V_2_O_5_ (**c**), and W–V (**d**) between the colored (−1 V) and bleached (+1 V) states at 700 nm for 1000 s; tests were performed in 1.0 M LiClO_4_/PC [82].

Prasad et al. [82] applied a one-step electrochemical co-deposition procedure to deposit a W–V mixed metal oxide with a composite thin-film structure consisting of WO_3_ and V_2_O_5_. The electrochemical energy storage and electrochromic parameters of the deposited thin films with different amounts of WO_3_ and V_2_O_5_ were investigated. In the films of the W–V mixed composites with WO_3_ and V_2_O_5_ oxides at a ratio of 1:1, the electrochromic properties were measured, and the findings included a fast coloration response of 4.9 s, an optimal optical contrast of 60%, and the highest coloration efficiency of 61.5 cm^2^/C; see Figure 8. Furthermore, for an electrochemical energy storage application, a maximum unit surface capacitance of 38.75 mF/cm^2^ at an applied current of 0.5 mA/cm^2^ was reached, and a capacitive retention of 78.5% was displayed, even after 5000 charge/discharge cycles. The enhanced characteristics for both electrochemical energy storage and electrochromic applications were associated with the (a) fast charge transfer kinetics; (b) unique morphology containing more activity in both cases, including WO_3_ and V_2_O_5_; and (c) redox behavior of both metal ions present in the synthesized W–V mixed metal composite.

#### 2.2.6. V_0.50_Ti_0.50_O_x_

Burdis et al. [83] applied RF sputtering from metallic targets for the deposition of thin layers of V_0.50_Ti_0.50_O*_x_*. They used this film as a potential counter-electrode to study its characteristics as an electrochromic device. They found that the film could reversibly store relatively large amounts of charge, and it was slightly yellow-looking in its transmission while displaying a reasonably low electrochromic coloration efficiency. The possible electrochemical reactions of V_0.50_Ti_0.50_O*_x_* were found to be simple; in fact, they were rather the same as that of WO_3_. For these reasons, they found that this material could be considered almost equivalent to use in a variable-transmission device. The charge capacity was found to be 60 mC/cm^2^ for a film thickness of 300 nm. The authors planned to further study the electrochromic coloration of these films up to high levels of charge insertion and to determine the effect of repeated cycles of charging and discharging on the lifetime of such structures.

Marcel et al. [84] overlaid two tungsten and vanadium–titanium oxide thin films to decrease the blue absorption of vanadium oxide prepared using the roll-to-roll radiofrequency sputtering method. In order to produce flexible devices that were adaptable for eyewear purposes, an ITO-coated mylar substrate was used. Tungsten oxide was applied as a working electrode, while the examined counter-electrodes were vanadium–titanium oxide mixtures. The electrolyte used for both electrodes was a polymer gel lithium ionic conductor made of a lithium salt (LiTFSI) dissolved in PC and incorporated within a photopolymerized acrylate matrix. They studied the electrochromic properties of the counter-electrode at four different atomic ratios of titanium in the range of 0–100%, increasing in steps of 25%. A blue-shift effect in the transmittance spectra of the as-deposited films was measured as the titanium amount was increased. The in situ optical characteristics were investigated by cycling the potential in the range of 1.5–4 V, and the sample with an equal proportion of vanadium and titanium displayed a notable neutrality of coloration. Complete devices were prepared with different Ti/V ratios of 0, 1:3, 1:1, and 3:1 in the counter-electrode. The film thickness of tungsten oxide was secured at 300 nm, while the thicknesses of the vanadium–titanium oxide films was set based on their respective electrochemical capacities. The obtained electrochromic characteristics, together with their cycling durability and response times, were evaluated in order to find the optimal vanadium–titanium composition.

#### 2.2.7. WO_3_–MoO_3_

Only in a few cases have the properties of mixed tungsten and molybdenum oxides been studied, despite the fact that they are the most studied material for electrochromic devices. Prameela and coworkers [85] studied these mixtures, but only in a small number of compositions between 0 and 100% in 20% steps.

Chaichana et al. [16] prepared molybdenum/tungsten trioxide (MoO_3_/WO_3_) electrochromic films from a peroxotungsten acid (PTA) solution on an indium-doped tin oxide (ITO) glass substrate using sol–gel and dip-coating methods in compositions of 15%, 30%, and 50%. The effect of the MoO_3_ ratio on the electrochromic, optical, and microstructural properties of the films was studied by applying cyclic voltammogram, spectrophotometry, AFM, XPS, XRD, and SEM. The 30%-MoO_3_-doped WO_3_ film showed the best diffusion coefficient, indicating that it had the best electrochemical properties. The MoO_3_/WO_3_ composition of 30% was found to cause the largest color difference between the bleached and colored states in comparison with the other compositions (44%). The maximum diffusion coefficient was approximately 3.4 × 10^−11^ cm^2^/s.

Faughnan and Crandall [86] concluded that the electrochromic optical absorption of mixed MoO_3_/WO_3_ amorphous films occurs at shorter wavelengths than in the case of pure oxides. The dependence of wavelength shifts as a function of MoO_3_ concentration and optical density was measured, but only with compositions of *x*-5% MoO_3_, *x*-30% MoO_3_, and *x*-75% MoO_3_. They explained their data using the intervalence charge transfer model, according to which electrons were at a level that was 0.7 eV higher in W^6+^ ions than electrons trapped in Mo^6+^ ions [87]. WO_3_ films are not the best for screens, as their peak absorption is about 885 nm, while our eyes are most sensitive at 550 nm. The maximum absorption peak of mixed oxides is at 575 nm. That is, less charge needs to be injected into a mixed oxide film to achieve a better contrast ratio, since the absorption peak is more in line with the reaction of the eye.

Hamelmann et al. [88] studied the electrochromic and other properties of pure and 0.5:0.5 WO_3_/MoO_3_ layers made using the sol–gel technique. Raman spectrometry studies showed that the WO_3_ and mixed films were generally amorphous, while the pure MoO_3_ film proved to be crystallized. After annealing at 270 °C, the films showed good electrochromic behavior. All the films fulfilled the requirements for electrochromic device applications, since their coloration efficiencies ranged between 60 and 120 cm^2^/C. 

Arvizu et al. [89] studied the electrochromicity of mixed W–Mo oxides deposited through DC magnetron co-deposition. They used eight different compositions ranging from 1:0 to 0.7:0.3. In particular, the cyclic durability and spectral properties of the samples were studied. It was observed that the injected charge was greater than the charge extracted in the first cycle. The observed effect was increased with a higher Mo content. The change in the injected electric charge was attributed to trapped and accumulating Li ions and increasingly difficult intercalation. Every film acquired a characteristic yellowish color.

In cyclic durability measurements, the peak of absorption shifted towards shorter wavelengths compared with pure WO_3_, and the layers became grayish in the colored state. The authors found that mixed oxides were better for "smart windows" because they could give a more neutral dark state.

**Figure 9:**
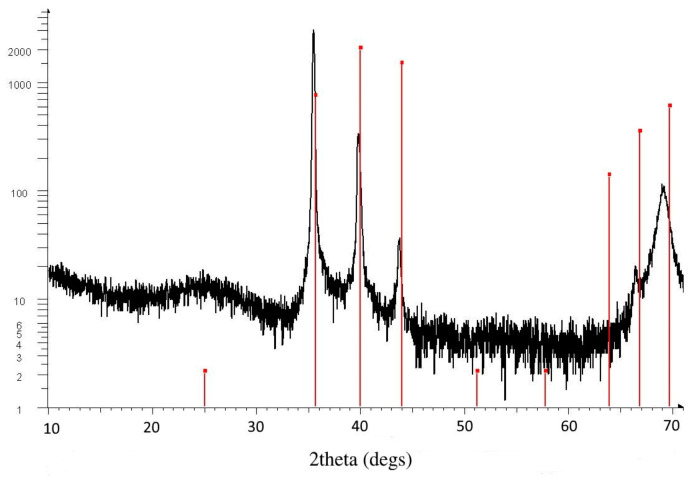
An example of several XRD measurements showing one significant broad peak in the 20–30° region with only an amorphous microstructure in the electrochromic layer. The vertical red lines show the calculated positions of beta tungsten, which was a thin (approximately 100 nm) layer below the EC film. The vertical red lines show the calculated positions of monoclinic, triclinic, and orthorhombic WO_3_ and hexagonal and orthorhombic MoO_3_ peaks. There are no traces of crystalline WO_3_ or MoO_3_ materials in the layers [90].

Labadi et al. [90] fabricated a combinatorial Mo*_x_*W_1−*x*_ oxide thin-film sample (where 0 < *x* < 1) through reactive magnetron sputtering onto ITO-coated glass and determined the optimal composition for the best EC efficiency. A continuous composition range was deposited in an experiment on a single substrate using a combinatorial process, and the samples were used to realize the entire composition range of the MoO_3_–WO_3_ system. For the same sputtered layers, a non-contact composition determination method was refined through spectroscopic ellipsometry. The layers proved to be amorphous according to the XRD and SEM measurements; see Figure 9. Four Si probes were examined—one from the “W side”, two from the “mixed part”, and one from the “Mo side”. It was found that the layers were highly amorphous. One example (from the mixed part) of the XRD measurements is shown in Figure 9. Only one significant broad peak in the 20–30° region could be seen as a sign of an amorphous film. The crystalline peaks at higher angles could be identified as the peaks of pure cubic (beta) tungsten, which was sputtered under the WO_3_ and MoO_3_ layers. The broad peak near 70° was from the silicon substrate. The vertical red lines show the calculated positions of beta tungsten, which was a thin (approximately 100 nm) layer below the EC film. The vertical lines show the calculated positions of monoclinic, triclinic, and orthorhombic WO_3_ and hexagonal and orthorhombic MoO_3_ peaks. There were no traces of crystalline WO_3_ or MoO_3_ materials in the layers. 

**Figure 10:**
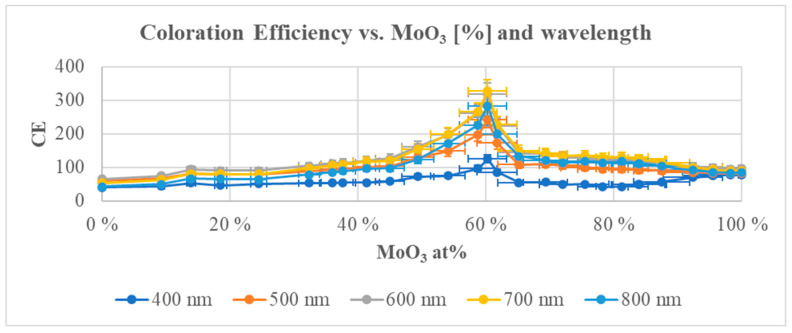
A significant CE maximum at 60% Mo content is shown. This maximum increased towards the red end of the spectrum in the visible spectral range of 400–800 nm [90].

By applying electric current through the layers and simultaneously measuring the layer transmittance, the electrochromic properties of the oxide mixtures were determined. Electrochromic redox reactions were investigated in an organopropylene carbonate electrolyte cell in a standard three-electrode arrangement. The coloration efficiency data were determined by measuring the transmission in the spectral range of 400–800 nm. The CE data showed a significant maximum of around 60% MoO_3_. It was possible to determine the location of the maximum with an accuracy of 5% due to the combinatorial approach; see Figure 10.

#### 2.2.8. TiO_2_–MoO_3_

Shrestha et al. [91] anodized Ti–Mo mixtures to produce TiO_2_–MoO_3_ composite oxide nanotubes with variable compositions. These nanotube layers showed significantly better electrochromic color contrast than that of pure TiO_2_ nanotubes. Self-assembling binary oxide nanotube layers were made. A Ti–Mo (7 wt%) alloy plate was polished to a mirror-smooth consistency and used as a working electrode in a conventional anodizing cell. It consisted of a conventional three-electrode system consisting of an Ag/AgCl (3 M KCl) reference electrode and a Pt mesh as counter electrodes. The reflective color contrast of amorphous Ti–Mo nanotubes was 2.5 times higher than that of amorphous titanium oxide nanotubes with equal charge density.

Ezhilmaran and Bhat [92] prepared an electrochromic device from a bilayer electrode with nanoparticulate TiO_2_ on the substrate and MoO_3_ nanograins in the top layer. The TiO_2_, MoO_3_, and TiO_2_/MoO_3_ layers were made using spin coating on conducting fluorine-doped tin oxide (FTO) substrates. Compared with data in the literature, the electrode behaved better in terms of its higher current density and charge storage capacity, as well as speed. They obtained a 40% color contrast, ~2 s switching response, and 72.5 cm^2^/C CE. 

**Figure 11:**
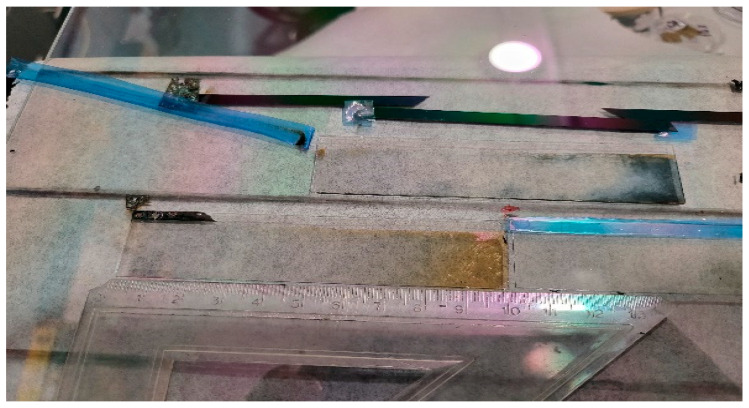
TiO_2_–MoO_3_ layers on ITO-covered glasses after electrochromic experiments [93].

**Figure 12:**
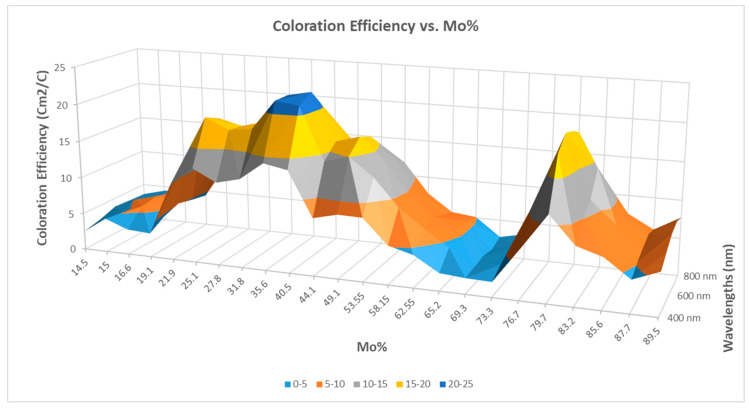
The coloration efficiency vs. Mo% of TiO_2_–MoO_3_ for wavelengths from 400 to 800 nm [93].

Ismaeel et al. [93] studied combinatorial mixed layers of a titanium oxide and molybdenum oxide (Mo*_x_*Ti_1−*x*_) thin-film system, and the optimal composition (where 0 < *x* < 1) was determined for electrochromic purposes. The combinatorial mixed layers were prepared in one step using a reactive magnetron-sputtering method. The layers were deposited onto ITO-covered glass to determine the optimal composition for the best coloration efficiency (CE). The CE was determined in a transmission electrochemical cell. To determine the position-dependent composition, scanning electron microscopy (SEM) with energy-dispersive X-ray spectroscopy (EDS) was applied; see Figure 11 and Figure 12. The two maximum peaks in the CE results can be clarified as follows: during the deposition process, the Ti-rich side was obviously subjected to a higher temperature, so the Ti-rich oxide was considered polycrystalline, while the Mo-rich side, which was the oxide, was still nanocrystalline or amorphous [93].

The results obtained by Habashyani et al. [94] using radio-frequency magnetron sputtering (RFMS) were consistent with those recently published in [93]. They made MoS_2_ thin films using a non-doped and Ti-doped perpendicular nanowall design. Using these production parameters (O_2_ gas around 500 sccm, oxidation time of 45 min at 380 °C), their layers were thermally oxidized to α-MoO_3_. The samples were labeled as follows: undoped MoO_3_ -> MBO, Ti:MoO_3_ with 20 W RF power -> MTO20, Ti:MoO_3_ with 30 W RF-power -> MTO30, Ti:MoO_3_ with 40 W RF-power -> MTO40. A condensed nanowall was manufactured through Ti doping. In the visible region, and with the increase in the Ti concentration inside the range of coloring potential from −0.2 to −0.45 V, optical modulation (OM) was optimized. This is a proportionally low working voltage that characterizes energy-saving electrochromic materials. The samples showed that 52.2% OM was the highest for the Ti-doped material, and 37.6% OM was the highest for the undoped MoO_3_ (MTO40) (MTO) (47.8% at λ = 700 nm and 25.7% at λ = 550 nm, respectively); the applied potential was −0.45 V. Moreover, 4.7, 4.1, 6.2, and 2.9 s were the coloring times for the MBO, MTO20, MTO30, and MTO40 samples, respectively, while 3.1, 1.4, 1.1, and 1.2 s were the bleaching durations for the same samples. The MBO sample was the non-doped one, while the Mo/Ti ratio was 80 in the MTO20 sample and 17 in the MTO30 sample; the lowest Mo/Ti ratio was 7 in the MTO40 sample. At visible wavelengths, this thin film presented the best OM and the best response time for coloring.

Even though the highest Ti-doped thin film destroyed the MTO40 wall structure, the MTO30 and MTO20 samples could be better implemented at longer wavelengths with higher coloration efficiency (CE) and optical modulation (OM). The authors drew the following conclusions: Ti doping was advantageous for electrochromic parameters such as the response times during the bleaching and coloring of MoO_3_, OM, and CE. These electrochemical attributes of Ti-doped MoO_3_ reveal the fitness of these materials for the application of this type of equipment.

#### 2.2.9. TiO_2_–SnO_2_

**Figure 13:**
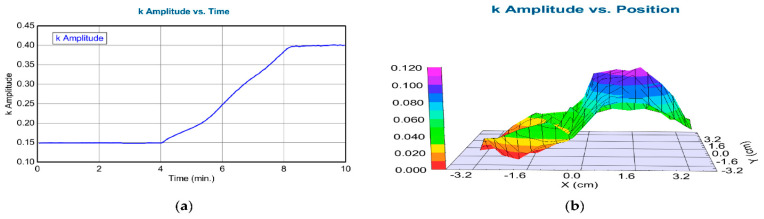
(**a**) The imaginary part of the refractive index (*k* amplitude) as a function of time for highly conductive Si in a liquid cell during coloration (time-scan, simple two-layer Cauchy model). From 0 to 4 min, there was low absorption, but from 4 to 8 min, there was an increase in absorption; (**b**) map of the *k* parameter after coloration (simple one-layer Cauchy model) [95].

Ismaeel et al. [95] determined the optimal composition of reactive magnetron-sputtered combinatorial mixed layers of titanium oxide and tin oxide (TiO_2_–SnO_2_) for electrochromic purposes. They mapped and determined the composition and optical parameters using spectroscopic ellipsometry (SE). Different optical models, such as the 2-Tauc–Lorentz multiple oscillator model (2T–L) and the Bruggeman effective medium approximation (BEMA), were used to obtain the composition maps and the thickness of the sample. Scanning electron microscopy (SEM) with energy-dispersive X-ray spectroscopy (EDS) was used to verify the SE results. They also compared the performance of various optical models. It was shown that in the case of molecular-level mixed layers, 2T–L was better than an EMA-based optical model. Using SE, Figure 13 shows a map of EC efficiencies for TiO_2_–SnO_2_ mixed metal oxides that were deposited using reactive sputtering.

The coloration process was followed by SE in situ at the center point of the mixed metal oxide–highly conductive Si sample. The authors mapped the colorized layer using a simple one-layer Cauchy dispersion optical model after the coloration process. For the Cauchy model, the *k* amplitude (extinction) parameter has been considered a good indicator of the coloration efficiency, as shown in Figure 13b. The maximum *k* value showed that the optimal composition was 30% TiO_2_ and 70% SnO_2_; see Table 2.

#### 2.2.10. WO_3_–MoO_3_–V_2_O_5_

Asymmetric and symmetric EC cells were studied by Sato and Seino [96], who used vacuum-evaporated amorphous WO_3_–MoO_3_–V_2_O_5_ films in propylene carbonate and lithium perchlorate solutions. They used the reflection spectra of symmetric cells and transmission spectra of asymmetric cells at a certain applied voltage. Compared with pure WO_3_ and V_2_O_5_ films, dark displays were obtained using WO_3_–MoO_3_ films when negative voltages were applied, and the contrast ratio was improved. V_2_O_5_–MoO_3_ films gave yellow and bluish displays, and reddish displays were obtained with WO_3_–MoO_3_ films. The authors found improved EC properties at a composition of 60% W0_3_ and 40% V_2_O_5_, giving colors between brown and yellow-green.

#### 2.2.11. SnO_2_–ZnO

Labadi et al. [97] conducted an electrochromic study to optimize the composition of mixed zinc oxide and tin oxide (ZnO–SnO_2_) layers produced through reactive magnetron sputtering. Pure metal targets of zinc and tin were placed separately, while indium–tin oxide (ITO)-coated glass samples and silicon probes on a glass holder were moved beneath the two targets within a reactive argon–oxygen (Ar–O_2_) atmosphere. This combinatorial approach allowed for the deposition of all possible compositions (ranging from 0% to 100%) within a single sputtering cycle.

The resulting SnO_2_–ZnO binary system spanned the entire compositional spectrum. The coloration efficiency (CE) of the mixed oxide films was determined by simultaneously measuring the layer’s transmittance in a conventional three-electrode setup and applying an electric current using organic propylene carbonate electrolyte cells. The optical properties and compositional data were assessed and mapped using spectroscopic ellipsometry (SE). To validate the SE results, scanning electron microscopy (SEM) and energy-dispersive X-ray spectroscopy (EDS) analyses were performed.

**Figure 14:**
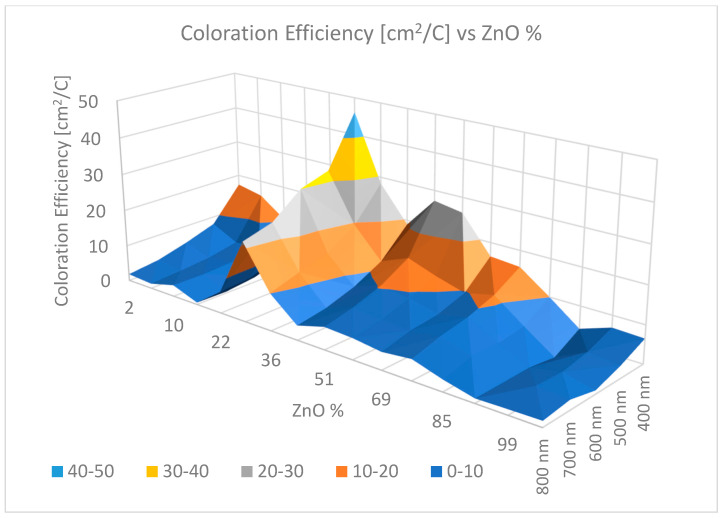
Three-dimensional diagram of the CE data of SnO_2_–ZnO vs. Zn % for wavelengths from 400 to 800 nm in the visible spectral range [97].

The electro-optical measurements revealed a peak in CE at approximately 29% ZnO, as illustrated in Figure 14. The accuracy of this combinatorial method was within 5%.

**Figure 15:**
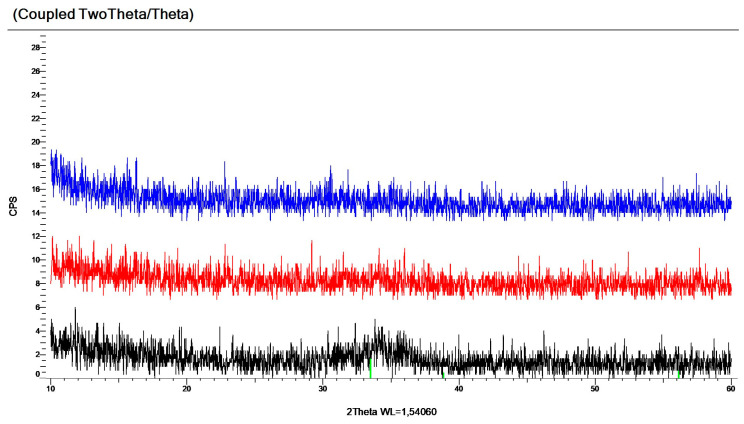
Examples of XRD measurements from the “Sn side” (upper), one from the “mixed part” (middle), and one from the “Zn side” (lower). One can see only small and wide peaks (between 33 and 37 deg) in the lower diffractogram, showing traces of small ZnO nanocrystallites (with diameters of less than 10 nm) [97].

The microstructures of the layers were examined using XRD in three characteristic zones: one from the “Sn side”, one from the “mixed part”, and one from the “Zn side”. It was found that the “Sn side” and the center (mixed) parts were fully amorphous, but the “Zn side” contained some nanocrystalline fractions. Examples of the XRD measurements are shown in Figure 15.

#### 2.2.12. Ir–Ta Oxide

Seong Uk Yun et al. [98] used a reactive co-sputtering system to deposit iridium tantalum oxide thin films. They characterized the prepared IrTaO*_x_* thin-film layers using in situ transmittance measurements, transmission electron microscopy (TEM), X-ray photoelectron spectroscopy (XPS), chronocoulometry, and electrochemical impedance spectroscopy (EIS). By increasing the tantalum composition, they detected an increase in the oxidized iridium in IrTaO*_x_*. According to the authors, the high transmittance modulation for the IrTaO*_x_* thin films was caused by the proton conductivity of tantalum. Their results showed that the Ir_33_Ta_67_ oxide thin film had a response time of 1.4 s, a coloration efficiency of 20 cm^2^/C, and an ion diffusion coefficient of 5 × 10^−9^ cm^2^/s. Based on their fast response time, these enhanced IrTaO*_x_* thin films are expected to be a candidate for electrochromic materials.

### 2.3. Newest Materials of Interest in EC Applications

Recent state-of-the-art developments in the field of ECDs focus on two main fields: multicolor displays and flexible devices. Chemically modified oxide-based electrochromic materials offer the possibility of making color ECDs. For example, phosphonate-modified mesoporous TiO_2_ films displayed a range of visible colors, while ferrocene electron sources blended in ion gel electrolytes allow the possible preparation of multicolor ECDs with low steady-state power consumption. However, most existing multicolor ECDs still lack a wide color gamut, which is necessary to achieve full-color displays [99].

In the field of flexible ECDs, graphene, silver nanowires, and carbon nanotubes are used as flexible electrodes. The roll-to-roll process allows the deposition of these materials on flexible substrates with substantially increased throughput. However, in flexible ECDs, the problem of uniformity across large surfaces, especially under repeated deformations and mechanical stress, is still far from being solved.

Recently developed organic polymers, metal supramolecular polymers, and metal–organic frameworks (MOFs) are regarded as the fifth generation of EC materials. These metallo-supramolecular polymers (MSPs) are widely studied for their good electrochemical and optical properties due to the electronic interactions between metals and ligands [100].

## 3. Conclusions

Many binary oxides have been studied as potentially promising EC materials. However, most of the studies have investigated only a few compositions. Some have only studied the effect of adding a single percentage value of a secondary material. Only a few examples can be found where a comprehensive investigation spanning the full compositional range between the component oxides was conducted. Notably, in most cases, mixed metal oxides have shown better EC properties than those of pure oxides.

- TiO_2_ nanorods/hybrid WO_3_ films exhibited impressive electrochemical characteristics; the diffusion coefficient of 1.8 × 10^−7^ cm^2^/s surpassed those of pure (WO_3_ and TiO_2_) nanorods.
- Nd–Mo-co-doped SnO_2_/α-WO_3_ ECs revealed up to 90% visible-light transparency at λ = 600 nm in comparison with conventional SnO_2_/α-WO_3_ ECs after up to 1000 trials of volatile cycles. There was a 59% drop in electrochromic functionality with respect to the undoped device after up to 1000 reversible cycle tests. Moreover, these doped samples displayed a shorter switching time (31% of the undoped value) and high coloration efficiency (~200 cm^2^/C).
- The EC performance of 1.2 wt% antimony-doped tin oxide nanoparticles in the WO_3_ EC film was better in terms of the CE value (48 cm^2^/C) and the switching time (2.4 s for the bleaching time and 5.4 s for the coloration time).
- The addition of 40% Ni to W oxide enhanced the coloration efficiency to 80 cm^2^/C.
- Higher surface roughness, clear optical modulation (41%), and high coloration efficiency (90 cm^2^/C for red) were detected in WO_3_–Ag thin films. In another experiment, the W_0.91_Ag_0.09_O_3−δ_ thin layer had a faster switching time with a higher coloration efficiency of 67 cm^2^/C than the WO_3−δ_ thin layer, which had an efficiency of 59 cm^2^/C. However, the transmittance modulation in the W_0.91_Ag_0.09_O_3−δ_ thin film was worse than in the other films.
- In the V_2_O_5_(85%)–WO_3_(15%) film, the electrochemical durability of the samples was found to be stable for up to 1000 cycles with 49 cm^2^/C. In W–V films mixed at a ratio of 1:1, the electrochromic properties were measured; the findings included a fast coloration response of 4.9 s, an optimal optical contrast of 60%, and the highest coloration efficiency of 62 cm^2^/C. Furthermore, for an electrochemical energy storage application, a maximum unit surface capacitance of 39 mF/cm^2^ at an applied current of 0.5 mA/cm^2^ was reached, and the material displayed a capacitive retention of 78.5%, even after 5000 charge/discharge cycles.
- The CE data showed a significant maximum for the magnetron-sputtered WO_3_(40%)–MoO_3_(60%) composition with values of 200–300 cm^2^/C in the visible range.
- Magnetron-sputtered SnO_2_(71%)–ZnO(29%) revealed CE values of 30–40 cm^2^/C as a maximum in the case of SnO_2_–ZnO mixtures.

Among these experiments, we found the WO_3_- and MoO_3_-based mixtures to be most promising, especially the magnetron-sputtered amorphous WO_3_(40%)–MoO_3_(60%) composition with a CE of 200–300 cm^2^/C. However, it is important to study the other EC properties (cycling stability, switching time, modulation depth) and the scale-up possibilities or other (physical or chemical) industrial preparation methods. The mixed oxide materials reported in this review represent room for development (and even commercialization) in the oxide-based EC device market. Provided that the proper optimized composition is determined in preliminary studies, further scaling up does not require fundamental changes in technology. For example, a reactive sputtering deposition process for WO_3_ layers can be adapted to the deposition of an amorphous WO_3_(40%)–MoO_3_(60%) mixed oxide by applying a proper alloy target. Better comprehension and the enhanced preparation of mixed metal oxides would provide better opportunities for economically feasible mass production. The potential economic impact of developing thin films with selected properties depends on the capabilities for the preparation of highly accurate test samples to locate the compositions with the optimal properties. The ideal compositions determined from combinatorial samples can, in turn, be used in industrial applications.

The application of mixed metal oxides is among the options that are considered environmentally friendly in fields such as energy saving and economic sustainability. The transition of the production of these materials to the industrial scale could enhance productivity and efficiency. This would contribute to reducing the extra heating absorption of buildings by applying thin-film coatings using materials similar to those presented in this study in a smart window. Finally, an additional outstanding feature of these material systems is their non-toxicity.

## Figures and Tables

**Table 1:** Electrochromic properties of WAg_-0_ and WAg_-75_ thin films in a 0.5 M solution of LiClO_4_-PC at 632.8 nm [79].

Sample	DC Voltage Steps (V)	T_c_ (%)	T_b_ (%)	ΔT (%)	CE (cm^2^C^−1^)	τ_C_ (s)	τ_B_(s)	Γ(λ) (cm^2^C^−1^s^−1^)
WAg_-0_	+1.0 to −1.0	45.15	82.18	37.03	71.9	4.2	11	9.46
WAg_-75_	+1.0 to −1.0	43.09	83.68	40.59	90.2	3.9	8.9	14.09

**Table 2:** The *k* amplitude vs. position at the center line after colorization in the dry state [95].

X (cm)	k Amplitude (Error ± 0.005)
−3.5	0.0002
−3	0.0025
−2.5	0.044
−2	0.004
−1.5	0.015
−1	0.025
−0.5	0.056
0	0.041
0.5	0.092
1	0.105
1.5	0.075

## Data Availability

The data that support the findings of this study are available in the public domain via the References.
